# Enhanced Postsurgical Cancer Treatment Using Methacrylated Glycol Chitosan Hydrogel for Sustained DNA/Doxorubicin Delivery and Immunotherapy

**DOI:** 10.34133/bmr.0008

**Published:** 2024-03-23

**Authors:** Hee Seung Seo, Jun-Hyeok Han, Jaesung Lim, Ga-Hyun Bae, Min Ji Byun, Chi-Pin James Wang, Jieun Han, Juwon Park, Hee Ho Park, Mikyung Shin, Tae-Eun Park, Tae-Hyung Kim, Se-Na Kim, Wooram Park, Chun Gwon Park

**Affiliations:** ^1^ Department of Biomedical Engineering, SKKU Institute for Convergence, Sungkyunkwan University (SKKU), 2066, Seobu-ro, Jangan-gu, Suwon, Gyeonggi 16419, Republic of Korea.; ^2^ Department of Intelligent Precision Healthcare Convergence, Institute for Convergence, SKKU, 2066, Seobu-ro, Jangan-gu, Suwon, Gyeonggi 16419, Republic of Korea.; ^3^ Department of Integrative Biotechnology, College of Biotechnology and Bioengineering, SKKU, 2066, Seobu-ro, Jangan-gu, Suwon, Gyeonggi 16419, Republic of Korea.; ^4^ Department of MetaBioHealth, SKKU Institute for Convergence, SKKU, 2066, Seobu-ro, Jangan-gu, Suwon, Gyeonggi 16419, Republic of Korea.; ^5^ Institute of Biotechnology and Bioengineering, College of Biotechnology and Bioengineering, SKKU, 2066, Seobu-ro, Jangan-gu, Suwon, Gyeonggi 16419, Republic of Korea.; ^6^ Department of Tropical Medicine, Medical Microbiology and Pharmacology, John A. Burns School Medicine, University of Hawai'i at Manoa, Honolulu, HI 96813, USA.; ^7^ Department of Bioengineering, Hanyang University, 222, Wangsimni-ro, Seongdong-gu, Seoul 04763, Republic of Korea.; ^8^ Department of Biomedical Engineering, Ulsan National Institute of Science and Technology, 50, UNIST-gil, Ulsan 44919, Republic of Korea.; ^9^ School of Integrative Engineering, Chung-Ang University, 84, Heukseok-ro, Dongjak-gu, Seoul 06974, Republic of Korea.; ^10^ Research and Development Center, MediArk Inc., 1, Chungdae-ro, Seowon-gu, Cheongju, Chungcheongbuk 28644, Republic of Korea.; ^11^ Biomaterials Research Center, Korea Institute of Science and Technology, 5, Hwarang-ro 14-gil, Seongbuk-gu, Seoul 02792, Republic of Korea.; ^12^ Biomedical Institute for Convergence, SKKU, 2066, Seobu-ro, Jangan-gu, Suwon, Gyeonggi 16419, Republic of Korea.

## Abstract

**Background:** Cancer recurrence and metastasis are major contributors to treatment failure following tumor resection surgery. We developed a novel implantable drug delivery system utilizing glycol chitosan to address these issues. Glycol chitosan is a natural adjuvant, inducing dendritic cell activation to promote T helper 1 cell immune responses, macrophage activation, and cytokine production. Effective antigen production by dendritic cells initiates T-cell-mediated immune responses, aiding tumor growth control. **Methods:** In this study, we fabricated multifunctional methacrylated glycol chitosan (MGC) hydrogels with extended release of DNA/doxorubicin (DOX) complex for cancer immunotherapy. We constructed the resection model of breast cancer to verify the anticancer effects of MGC hydrogel with DNA/DOX complex. **Results:** This study demonstrated the potential of MGC hydrogel with extended release of DNA/DOX complex for local and efficient cancer therapy. The MGC hydrogel was implanted directly into the surgical site after tumor resection, activating tumor-related immune cells both locally and over a prolonged period of time through immune-reactive molecules. **Conclusions:** The MGC hydrogel effectively suppressed tumor recurrence and metastasis while enhancing immunotherapeutic efficacy and minimizing side effects. This biomaterial-based drug delivery system, combined with cancer immunotherapy, can substantial improve treatment outcomes and patient prognosis.

## Introduction

Cancer, a global health issue, is a leading cause of death worldwide [1]. Over the past century, numerous therapeutic strategies have been developed for cancer, demonstrating positive effects in clinical applications [2,3]. However, conventional treatments such as chemotherapy and radiotherapy have limitations, such as severe side effects, high recurrence risk, and limited therapeutic impact, stressing the need to develop more efficient therapies [4,5]. Surgery is a common oncological intervention; however, the associated wound-healing process can inadvertently cause metastasis [6], accounting for 90% of cancer-related deaths [7]. Primary tumor recurrence after surgical intervention and metastasis correlate with poor outcomes, heightened local immunosuppression, and increased distant metastasis [8–10].

Cancer immunotherapy is an emerging groundbreaking approach in oncology. Unlike conventional therapies that directly target tumor cells, immunotherapy leverages the patient’s innate immune system to combat tumor cells [11]. This innovative strategy offers the potential to inhibit both metastasis and tumor recurrence effectively. Nevertheless, immunotherapy has encountered several significant obstacles. Historically, systemic administration has been the prevailing delivery method used in many preclinical investigations [12,13]. However, this approach activates the immune system systemically, potentially leading to life-threatening autoimmune diseases [14,15]. Furthermore, low drug delivery rates to tumor sites during systemic infusion decrease treatment efficiency by up to 30% [16].

To address and overcome these challenges posed by immunotherapy, extensive research is currently being conducted on various biomaterials [17–19]. Notably, implantable drug delivery systems have been researched, with hydrogels emerging as potential biomaterials for facilitating innovative drug delivery strategies [20,21]. Hydrogels have consistently demonstrated advantageous properties, such as low toxicity, high loading efficacy, stimuli responsiveness, and in situ gel-forming ability [22,23]. These favorable characteristics indicate that hydrogels can be a potential delivery approach that significantly enhances cancer/immune cell targeting, boosts immune responses, and reduces systemic toxicity and adverse side effects associated with immunotherapy [24–26].

Glycol chitosan (GC), the base constituent of hydrogels, is particularly advantageous for clinical and biological applications due to its low toxicity and immunogenicity, biocompatibility, and biodegradability [27,28]. Moreover, GC serves as a natural adjuvant, stimulating dendritic cell (DC) activation, which, in turn, enhances T helper 1 cell immune responses, macrophage activation, and cytokine production [29]. DCs are crucial in promoting effective antigen production and initiating T-cell-mediated immune responses, contributing to the tumor growth control [30,31].

Doxorubicin (DOX), established as a highly effective chemotherapeutic drug, induces immunogenic cell death (ICD) during cancer immunotherapy and inhibits nucleic acid synthesis in cancer cells [32,33]. DOX also induces ICD in various cancer cells through stimulation of DCs and macrophages and subsequently generates specific T cell responses [34]. Tumor cells undergoing ICD secrete damage-associated patterns (DAMPs), such as adenosine 5′-triphosphate (ATP) “danger signals” and calreticulin (CRT) “eat me” signals, promoting the recruitment and activation of antigen-presenting cells and activating cytotoxic T lymphocytes [35]. Thus, ICD can be synergized with the cancer immunotherapy [36,37].

In this study, we explored cutting-edge technologies to surmount the challenges of cancer recurrence and metastasis following tumor resection surgery by integrating cancer immunotherapy and chemotherapy within biomaterial-based drug delivery systems. Our approach entailed using methacrylated GC (MGC) and DOX to create a multifunctional MGC hydrogel system to amplify the anticancer immune response and instigate ICD. Nonetheless, DOX, a low-molecular-weight drug, poses substantial difficulties in encapsulation and controlled release within hydrogels featuring micrometer-sized pores. To address this issue, we used DNA/DOX complexes to effectively sequester DOX and regulate its release within the hydrogel matrix. Owing to its planar and aromatic structure, DOX can spontaneously intercalate into DNA molecules, a process facilitated by the flat surfaces of DNA base pairs [38]. The DNA complexed with DOX is still negatively charged, so it can electrostatically interact with the positively charged MGC hydrogel to control drug entrapment and release [39]. Moreover, DNA’s inherent biocompatibility as a natural biological material, ease of synthesis and modification, and extraordinary programmability render it an ideal candidate for drug delivery systems [40].

We investigated the immunotherapeutic efficacy of MGC hydrogel in the 4T1 breast cancer resection model. Multifunctional MGC hydrogel loaded with DNA/DOX complex was directly implanted into the surgical site after tumor resection to locally activate tumor-associated immune cells (e.g., cytotoxic T cells, CD4^+^ cells, and regulatory T cells). The DNA/DOX complex on the scaffold acted as an antigen source to eradicate tumor cells, mediating ICD of tumor cells to generate a host antitumor immune response, suggesting that MGC hydrogels can inhibit tumor recurrence and metastasis through the synergistic effect of immune activation and ICD induction via hydrogel and DOX. This approach ultimately aims to improve the efficacy of immunotherapy while minimizing side effects.

## Methods

### Materials

GC (catalog no. HY-135969; ≥80% deacetylation, ≥400 degree of polymerization, molecular weight = ~82 kDa) was obtained from MedChemExpress (NJ, USA). Glycidyl methacrylate was supplied by Tokyo Chemical Industry Co. Ltd. (Chuo-ku, Tokyo, Japan). Ammonium persulfate (catalog no. 7727-54-0), *N*,*N*,*N*′,*N*′-tetramethyl ethylenediamine (TEMED; catalog no. 110-18-9), lipopolysaccharide (LPS; catalog no. L2880), and DNA, sodium salt extracted from salmon testes (catalog no. D1626) were acquired from Sigma-Aldrich (Seoul, South Korea). DOX (hydrochloride salt, >99%) was purchased from LC Laboratories (MA, USA). Cyanine7 *N*-hydroxysuccinimide ester was provided by Lumiprobe (MD, USA). Recombinant mouse granulocyte-macrophage colony-stimulating factor (GM-CSF) was sourced from BioLegend (CA, USA). The D-Plus CCK cell viability assay kit was procured from Dongin Biotech Co. (Seoul, Korea). LDS 751 (catalog no. L7595), RPMI 1640 medium (catalog no. 22400, Gibco), fetal bovine serum (FBS; catalog no. 16000044, Gibco), and penicillin/streptomycin (catalog no. 15140163, Gibco) were obtained from Thermo Fisher Scientific (MA, USA).

### Animals and cell lines

All female BALB/c (6-week-old) mice were obtained from Orient Bio (Seongnam, Korea) and housed under pathogen-free conditions. All experiments were reviewed and approved by the Institutional Animal Care and Use Committee (IACUC) of Sungkyunkwan University School of Medicine (approval number: SKKUIACUC2023-02-16-1). This university is accredited by the guidelines of the National Research Council’s Guide for the Care and Use of Laboratory Animals. The L929 cell line (mouse fibroblast of connective tissue) was obtained from Korean Cell Line Bank (Seoul, South Korea). Bone-marrow-derived DCs (BMDCs) were used for in vitro tests. BMDCs were prepared from murine bone marrow cell cultured with GM-CSF (20 ng/ml) for differentiation. 4T1-Luc2 (a luciferase-expressing cells derived from the parent 4T1 cell line) cells were purchased from American Type Culture Collection. These cells were cultured in RPMI 1640 medium (RPMI 1640 is supplemented with 10% FBS, 1% penicillin/streptomycin, and 1% l-glutamine).

### Synthesis of MGC

GC was dissolved in 10 ml of distilled water (DW), and the pH was adjusted to 8.0 with 1 M NaOH to create a 2% (w/v) solution. This GC solution and glycidyl methacrylate were combined at a 0.75:1 molar ratio of glycidyl methacrylate to the amino groups in GC in DW. This mixture was allowed to react for up to 48 h. The reaction mixture was dialyzed using a molecular weight cutoff of 1 kDa of dialysis tubing using water for 2 d. The MGC solution was lyophilized to produce a white powder.

### Fabrication of MGC hydrogels

Synthesized MGC was dissolved in DW to obtain a concentration of 20 mg/ml. Ammonium persulfate, a radical source, was added to the polymer solution. The MGC solution was mixed with 50% (v/v) TEMED in phosphate-buffered saline (PBS), an initiator. The mixture was transferred to a polytetrafluoroethylene mold (diameter, 5 mm; thickness, 9 mm) and placed at −20 °C for 24 h. The fabricated scaffolds were sterilized with ethanol for 5 h, washed thrice with DW, and then lyophilized.

### Evaluating the gelation time of the MGC hydrogel

To assess the gelation time of the MGC hydrogel, it was prepared as described above. Subsequently, the times for the frozen state during cross-linking were varied at intervals of 6, 12, and 24 h to evaluate gelation.

### Nuclear magnetic resonance spectroscopy

^1^H nuclear magnetic resonance (NMR) spectra were obtained using a JNM-ECZ500R (JEOL Ltd.) 500-MHz spectrometer. Samples were prepared at a concentrations of 10 mg/ml in deuterium oxide. All chemical shifts were primarily and secondarily referenced to the heavy oxygen deuterium (HOD) peak and tetramethylsilane, respectively. Spectral data were collected using Delta v5.0 software. The degree of substitution (DOS) of methacrylate groups onto the GC backbone was calculated by the following equation [41]:

$$\text{DOS}=\frac{\left(I_{5.74}+I_{6.16}\right)/2\;}{I_{3.74}+I_{4.00}}\times 100\%$$

### Morphology and porous scaffold analysis

The porous structure of the scaffold was characterized using scanning electron microscopy (SEM; JSM-7000F, JEOL Ltd, Tokyo, Japan). Lyophilized scaffold samples were broken, sputter-coated with platinum, and examined at 10 kV. Scaffold pore size was determined using ImageJ image analysis software (ImageJ 1.48 bundled with 64-bit Java, National Institutes of Health). Pore diameter was expressed as the mean of 59 randomly selected samples from the SEM images.

### Swelling behaviors of the MGC hydrogel

The swelling ratio measurements of the hydrogels were conducted using a water absorptive approach on a predetermined amount of dried hydrogel samples. Initially, the freeze-dried hydrogel samples, preweighed and adjusted to the physiological pH of PBS (pH 7.4) and the tumor microenvironment pH (pH 6.5), were immersed in PBS. The swollen hydrogels were drawn from PBS at 24 and 48 h, gently blotted with filter paper to remove excess PBS, and then weighed using a precision microbalance. The percentage of water uptake was calculated using the following equation: swelling ratio (%) = (*Ws* − *Wd*)/*Wd* × 100.

Here, *Ws* and *Wd* represent the weight of the swollen MGC hydrogel and the weight of the dried MGC hydrogel, respectively.

### Rheological properties of MGC hydrogels

The rheological properties of the MGC hydrogel were determined through oscillation frequency sweep tests using Discovery Hybrid Rheometer 2 (TA Instruments, USA). All rheological measurements were conducted using an 8-mm parallel-plate geometry with a gap size of 3,000 μm. The storage (*G*′) and loss (*G*″) moduli were measured at room temperature under oscillation frequency sweeps (0.1 to 10 Hz, at 1% strain). To assess the mechanical strength modulation of the MGC hydrogel, various concentrations of MGC (1, 2, and 5 wt%) were formulated as mentioned above, and hydrogels were fabricated accordingly. The rheological properties of the MGC hydrogel were determined through oscillation frequency sweep tests using the Discovery Hybrid Rheometer 2 (TA Instruments, USA). All rheological measurements were conducted under the previously mentioned conditions.

### BMDC activation

BMDCs were seeded onto 96-well plates at a density of 1 × 10^5^ cells per well and cultured in RPMI 1640 medium. BMDCs were differentiated from bone marrow cells using GM-CSF (20 ng/ml) for 6 d. Immature BMDCs were treated with GC and MGC (25, 50, 100, 300, and 500 μg/ml), and molar ratio synthesized MGC hydrogel extracts for 24 h at 37 °C. BMDCs were also treated with LPS (50 ng/ml) as a positive control. To confirm the maintenance of immune-stimulatory capabilities even after forming the MGC hydrogel with non-cross-linked MGC, the dried MGC hydrogel (150 mg/kg) was extracted using RPMI 1640 medium at 37 °C for 7 d. To analyze the maturation markers of BMDCs, the cells were harvested and labeled with the following fluorescence-conjugated primary antibodies: BV510-conjugated Zombie aqua (catalog no. 423101, BioLegend, CA, USA; dilution, 1:100) for live cells; PerCp/Cy5.5-conjugated anti-CD11c (clone: N418, catalog number: 117328, BioLegend, CA, USA; dilution, 1:100), phycoerythrin (PE)/Cyanine7-conjugated anti-mouse CD40 antibody (clone: 3/23, catalog number: 124622, BioLegend, CA, USA; dilution, 1:100), Allophycocyanin (APC)-conjugated anti-CD80 (clone: 16-10A1, catalog number: 104714, BioLegend, CA, USA; dilution, 1:100), PE-conjugated anti-CD86 (clone: GL-1, catalog number: 105008, BioLegend, CA, USA; dilution, 1:100), and fluorescein isothiocyanate (FITC)-conjugated anti-major histocompatibility complex II (MHCII) (clone: 39-10-8, catalog number: 115006, BioLegend, CA, USA; dilution, 1:100) for DCs, respectively. The labeled cells were analyzed using a CytoFLEX flow cytometer (Beckman Coulter, CA, USA).

### Cytotoxicity

The cytotoxicity of MGC and MGC hydrogels was evaluated using the D-Plus CCK cell viability assay kit, following the manufacturer’s instructions. The dried MGC hydrogel (150 mg/kg) was extracted using RPMI 1640 medium for 5 d at 37 °C. Briefly, different concentrations of MGC and MGC hydrogel extracts were dissolved in serum-deficient RPMI 1640. L929 cells were seeded onto 96-well plates and incubated for 24 h (1 × 10^4^ cells per well). The cells were washed with PBS, and the dissolved sample solutions were added. After 24 h, the CCK-8 solutions were added and incubated for 1 h at 37 °C. To calculate cell viability, the absorbance of the well plates was measured at 450 nm using a microplate reader (BioTek Synergy HTX, VT, USA). The relative viability values were compared with the absorbance cells without treatment (control group).

### DNA/DOX complex preparation

To prepare the DNA/DOX complex, 1 mg of DOX was added to the 0.1% (w/v) DNA solution in DW. Next, the DNA/DOX mixture was incubated for 24 h at a constant temperature of 4 °C with continuous shaking. Following incubation, the mixture was dialyzed using a molecular weight cutoff of 1 kDa of dialysis tubing with water for 4 h and subsequently lyophilized. The DNA/DOX concentration in the dialysate was estimated using a microplate reader at a wavelength of 480 nm (BioTek Synergy HTX, VT, USA).

### In vitro release study

DNA/DOX complex (100 μg in DOX equivalent) dissolved in DW was loaded into MGC hydrogel by dropwise addition and lyophilization. To verify the encapsulation of the DNA/DOX complex, we quantified the amount of DNA/DOX not loaded into the hydrogel using microplate reader at a wavelength of 480 nm. The MGC hydrogel loaded with the DNA/DOX complex was evaluated through optical fluorescence imaging using a fluorescence-labeled organism bioimaging instrument system (Neo-Science, Suwon, Korea). For in vitro release study, the lyophilized MGC hydrogel loaded with DNA/DOX complex was placed in 2 ml of PBS in an incubator shaker at 37 °C under 90 rpm. The release medium was collected and replaced with fresh medium at selected time intervals. The amount of DNA/DOX complex released from the scaffolds was evaluated by measuring the absorbance using a microplate reader at a wavelength of 480 nm (BioTek Synergy HTX, VT, USA).

### In vivo release study

To assess the in vivo release profiles of the MGC hydrogel, hydrogels containing LDS 751-tagged DNA were surgically implanted into nontumor-bearing mice. For comparison, control solutions containing fluorescent-tagged DNA (LDS 751 solution) were administered at the incision sites. The release profiles were monitored by measuring fluorescence with the IVIS Spectrum In Vivo Imaging System (PerkinElmer, MA, USA).

### In vivo degradation study

The in vivo degradation of Cy7-labeled hydrogels was monitored using the IVIS Spectrum In Vivo Imaging System after surgically implanting the hydrogels into nontumor-bearing mice. Fluorescence imaging was performed weekly, and the data were analyzed using Living Imaging software (PerkinElmer, MA, USA).

### In vitro ICD assay

The 4T1-Luc2 cells were seeded at a density of 1 × 10^5^ cells in a 24-well culture plate. After a 24-h incubation, the cells were treated with various concentrations of DNA/DOX complex (in DOX equivalent) for 4 h. Following this treatment, the supernatant was removed, and the cells were washed thrice with PBS. The cancer cells were then resuspended in 500 μl of flow cytometry staining buffer and 1 μl of CRT antibody (clone 1G6A7, Novus Biologicals, CO, USA) and incubated at 4 °C for 1 h. Next, they were centrifuged, washed thrice with PBS, and resuspended in the flow cytometry buffer solution. Finally, the expression levels of CRT in the cancer cells were analyzed using flow cytometry (CytoFLEX, Beckman Coulter, Miami, FL, USA).

### Adenosine 5′-triphosphate

ATP release in response to DNA/DOX complex treatment was evaluated using ATP assay kits, respectively. Twelve hours after DNA/DOX complex treatment, culture medium supernatants were collected. ATP levels were measured with the ATP Bioluminescent Assay Kit (Sigma-Aldrich, Seoul, South Korea) according to the manufacturer’s instructions.

### In vivo tumor models and treatment

For the breast cancer resection model [21], 4T1-Luc2 cells (1 × 10^6^ cells) were orthotopically inoculated into the fourth mammary pad of BALB/c mice. Dried MGC hydrogel (150 mg/kg) was used in the experiment. Animal tests were conducted in 5 groups: (a) no hydrogel, (b) DOX solution (intraperitoneal injection at a dose of 5 mg/kg), (c) DNA/DOX complex solution (intraperitoneal injection at a dose of 100 μg in DOX equivalent), (d) 100 μg of DOX-loaded MGC hydrogel, and (e) DNA/DOX complex (100 μg in DOX equivalent)-loaded MGC-hydrogel-treated groups. Each group contained 5 animals. Surgery was performed when the tumor volume reached approximately 100 mm^3^. Mice were kept under anesthesia with 2% isoflurane, and the tumor was resected, intentionally leaving about 10% of tumor tissues intact. The MGC hydrogels were implanted in the site of the resultant cavity at the time of surgery, and the wound was closed with medical clips.

### In vivo bioluminescence and imaging

After surgery, the mice were monitored weekly for local tumor recurrence and distal metastasis using bioluminescence imaging. To perform the imaging, mice were intraperitoneally injected with d-luciferin (150 mg/kg), a substrate of Luc2, and, 10 min later, anesthetized with 2% isoflurane. Images were then acquired using the IVIS Spectrum In Vivo Imaging System.

### Evaluation of activated immune cells in vivo

Activated immune cell analysis was conducted in 3 groups: (a) no hydrogel, (b) DNA/DOX complex solution (intraperitoneal injection at a dose of 100 μg in DOX equivalent), and (c) DNA/DOX complex (100 μg in DOX equivalent)-loaded MGC-hydrogel-treated groups. Each group contained 5 animals. Ten days after the treatment procedure, the spleen and lymph node were harvested respectively. The tissues were mechanically dissociated, resuspended in fluorescence-activated cell sorting buffer (PBS with 2% FBS), and then filtered through a 70-μm Falcon cell strainer. The cells from tissues were collected, centrifuged at 250*g* for 5 min, and resuspended in fluorescence-activated cell sorting buffer. Erythrocytes of the tissues were lysed with red blood cell lysis buffer (ACK lysis buffer) for 5 min at 4 °C and then centrifuged at 250*g* for 5 min. Cell suspensions were prepared as described above, and then the cells were stained with the following antibodies: BV510-conjugated Zombie aqua (catalog no. 423101, BioLegend, CA, USA; dilution, 1:100) for live cells; PerCp/Cy5.5-conjugated anti-CD11c (clone: N418, catalog number: 117328, BioLegend, CA, USA; dilution, 1:100), APC-conjugated anti-CD80 (clone: 16-10A1, catalog no. 104714, BioLegend, CA, USA; dilution, 1:100), PE-conjugated anti-CD86 (clone: GL-1, catalog no. 105008, BioLegend, CA, USA; dilution, 1:100), and FITC-conjugated anti-MHCII (clone: 39-10-8, catalog no. 115006, BioLegend, CA, USA; dilution, 1:100) for DCs; BV785-conjugated anti-CD45 (clone: 30-F11, catalog no. 103149, BioLegend, CA, USA; dilution, 1:100), FITC-conjugated anti-T cell receptor β (TCRβ) (clone: H57-597, catalog: 109206, BioLegend, CA, USA; dilution, 1:100), BV605-conjugated anti-CD4 (clone: GK1.5, catalog no. 100451, BioLegend, CA, USA; dilution, 1:100), and BV650-conjugated anti-CD8 (clone: 53-6.7, catalog no. 100742, BioLegend, CA, USA; dilution, 1:100), and PE-conjugated anti-Foxp3 (clone: FJK-16s, catalog no. 25-5773-82, Thermo Fisher Scientific, MA, USA; dilution, 1:100) antibodies for T cells. The cells were then washed with PBS with 2% FBS twice and analyzed using flow cytometry (CytoFLEX S, Beckman Coulter) and the FlowJo software (BD Biosciences).

### Statistical analysis

Differences among groups were determined using analysis of variance (ANOVA) and Tukey’s method. Significance was set at *P < 0.05, **P < 0.01, ***P < 0.001, and ****P < 0.0001. All statistical analyses were performed using GraphPad Prism 8 (GraphPad Software, USA).

## Results

### Synthesis of MGC hydrogel

**Fig. 1.**
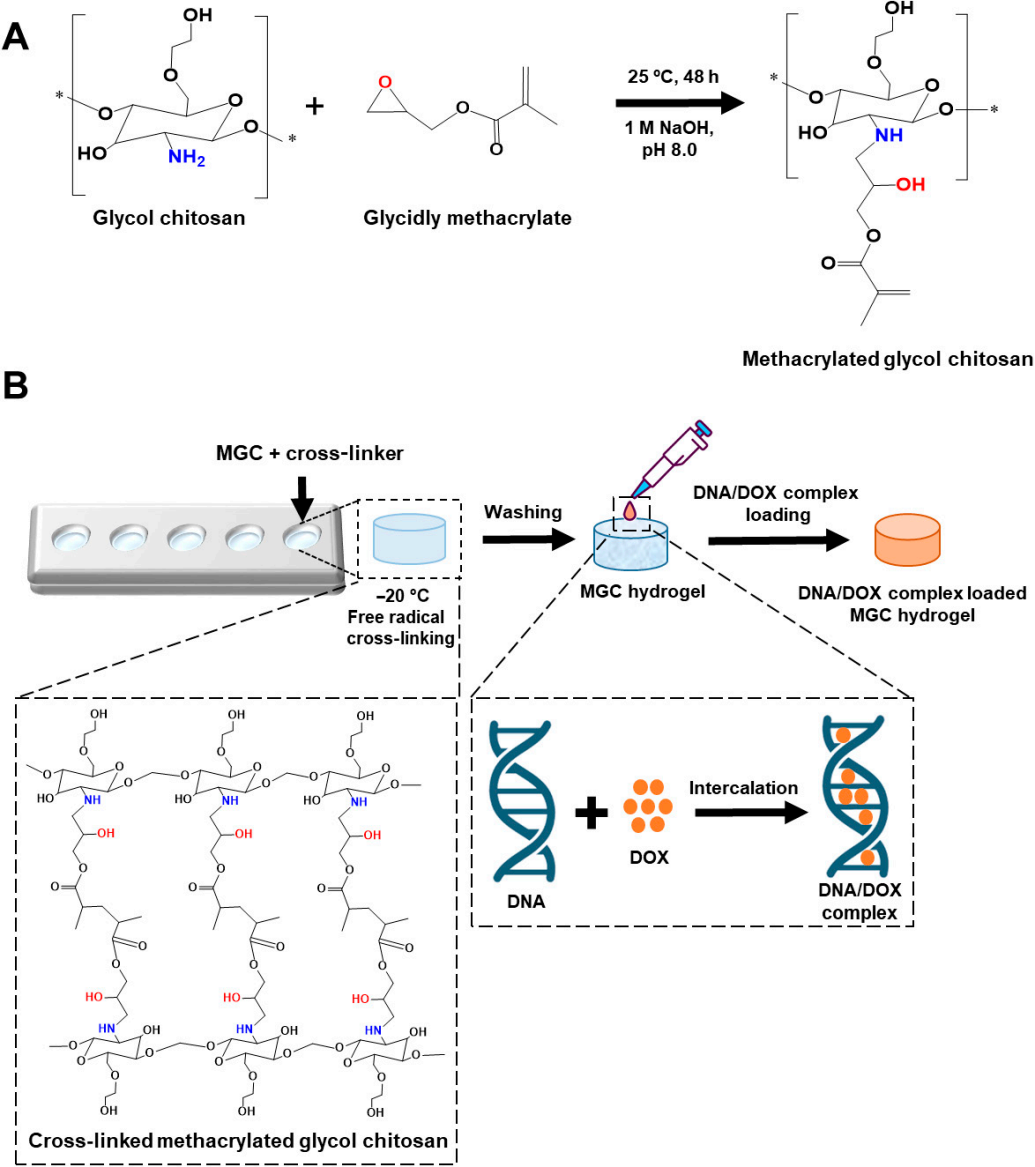
Synthesis of the multifunctional MGC hydrogel. (A) Synthetic process of MGC. (B) Schematic of the fabrication process of MGC hydrogel loaded with DNA/DOX complex.

The preparation of MGC hydrogel involved several steps entailing the following mechanisms. First, MGC was synthesized through a nucleophilic substitution reaction between glycidyl methacrylate and GC (Fig. 1A) [42]. Next, MGC was mixed with cross-linking precursors in a mold. Finally, the MGC hydrogel underwent cross-linking in a frozen state in the presence of a free radical initiator, with the cross-linking reaction occurring between the vinylic groups of methacrylate through free radical polymerization, and was subsequently freeze-dried (Fig. 1B). To assess the disparity in cross-linking between GC and MGC, we compared the outcomes of the cryogenic synthesis step and the subsequent ethanol washing step (Fig. S1). Notably, GCs lacking polymerization lost their structural integrity during ethanol washing. In contrast, MGC demonstrated the ability to form a stable hydrogel. When the MGC hydrogel was allowed sufficient time for cross-linking, specifically 24 h after freezing, a fully formed hydrogel with complete structural integrity was obtained. After freezing, hydrogels examined at 6 and 12 h did not allow sufficient time for proper cross-linking, resulting in incomplete forms when compared to the 24-h hydrogel. This was evident not only in terms of structural completeness but also in the observed differences in weight (Fig. S2).

**Fig. 2.**
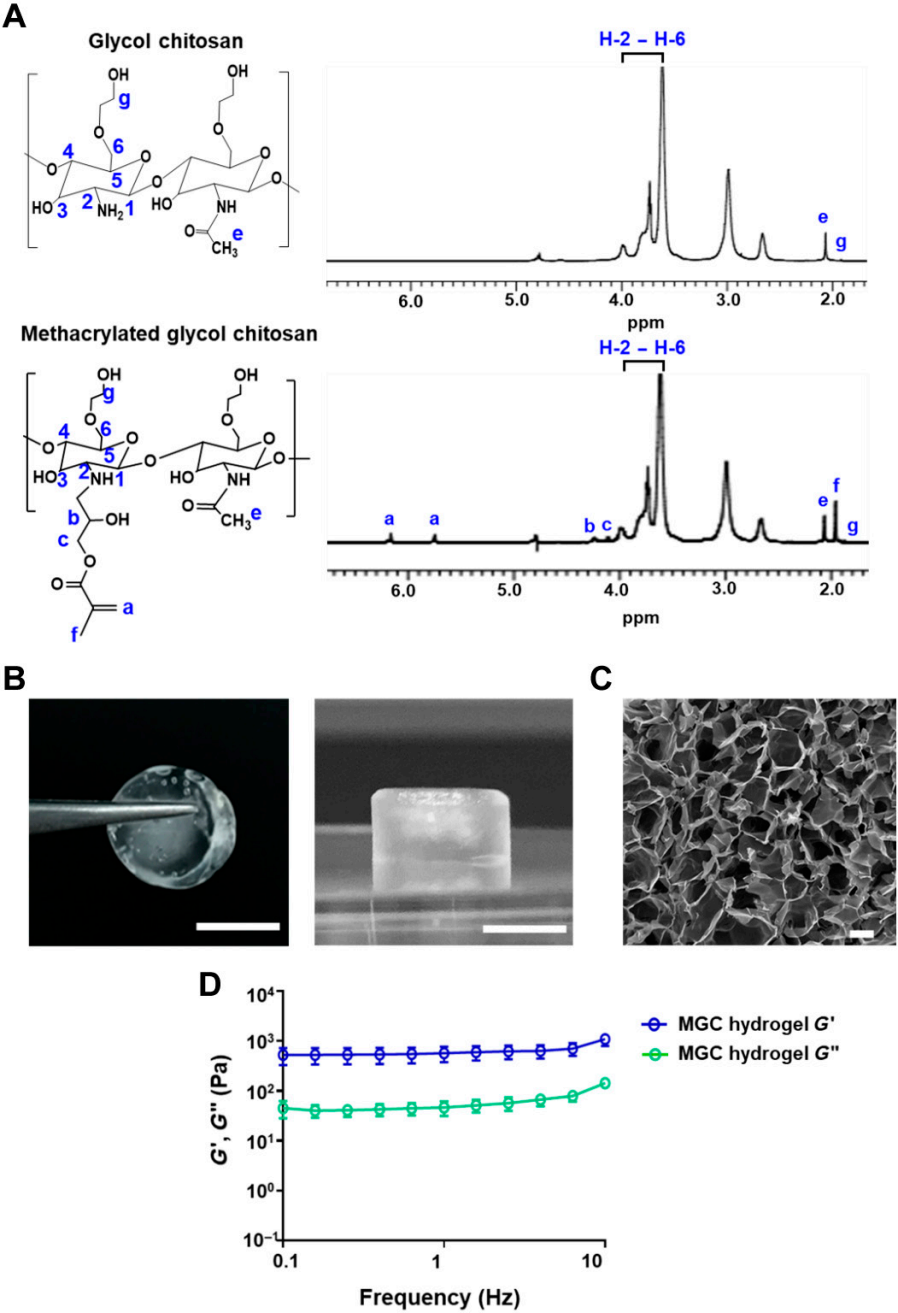
Characterization of multifunctional MGC hydrogel. (A) ^1^H NMR spectra of GC and MGC. (B) Optical images of MGC hydrogel scaffold. Scale bars, 5 mm. (C) SEM image of dehydrated MGC hydrogel. Scale bar, 100 μm. (D) Oscillation frequency sweep measurements of MGC hydrogels. The blue empty circles represent the storage modulus (*G*′), and the green empty circles represent the loss modulus (*G*″). Data are presented as means ± SD (*n* = 3).

To confirm the conjugation of GC and MGC, we determined their structures using ^1^H NMR (Fig. 2A). Successful methacrylation of GC was indicated by peaks at 5.74 and 6.16 parts per million (ppm) on vinyl carbon and 1.96 ppm on the methyl group in glycidyl methacrylate. Because of *N*-methacrylation, the peak area at 2.66 ppm was reduced (H-2 proton for the deacetylated residues), and a small peak appeared at 3.18 ppm, corresponding to H-2 for the *N*-methacrylated residue. There is also a concurrent reduction in the H-1 peak for the deacetylated residue (3.74 ppm), while the peak at 4.00 refers to acetylated residues. New peaks for *N*-methacylated residues were also observed, corresponding to H-1 at 4.11 and 4.24. The DOS of methacrylate groups onto the GC backbone was determined to be 5.57%. These findings confirmed that methacrylate had been successfully grafted onto the GC molecules. After cross-linking the hydrogel in a mold, the MGC hydrogel exhibited a cylindrical morphology with a diameter of approximately 9 mm and a thickness of around 7 mm (Fig. 2B). The photographic and SEM images of the MGC hydrogel synthesized through the cryogelation technique displayed an interconnected, macroporous structure with a pore size of 159.19 ± 69.86 μm (ranging from 37.62 to 322.49 μm) (Fig. 2C). This MGC hydrogel with macroporous structure was designed for releasing the loaded components in a localized and sustained manner. To assess the swelling property, we systematically investigated the swelling characteristics of the fabricated MGC hydrogel in both the general physiological environment (pH 7.4) and the tumor microenvironment (pH 6.5). When comparing the swelling ratios at 24 and 48 h, no significant difference in the hydrogel’s swelling behavior was observed between the general physiological environment and the specific tumor microenvironment (Fig. S3). To estimate the mechanical durability of the MGC hydrogels, we measured the storage modulus (*G*′) and loss modulus (*G*″) of the fabricated hydrogels while changing the frequency from 0.1 to 10 Hz under 1% shear strain (Fig. 2D). The storage modulus of the MGC hydrogels (570.8 Pa at 1 Hz) was constantly higher than their loss modulus (46.4 at 1 Hz) at all frequency ranges, indicating the high mechanical strength, elasticity, and high flexibility of the MGC hydrogel. We achieved precise control over the mechanical strength of the MGC hydrogel by meticulously adjusting the concentration of methacrylate GC (Fig. S4). The MGC hydrogel formation was challenging at 1 wt% of methacrylate GC, and we observed an increase in storage modulus values as the concentration increased. This highlights the capability of adjusting the concentration of methacrylate GC to modulate the mechanical strength of the hydrogel.

### DC activation by GC, MGC, and MGC hydrogel

**Fig. 3.**
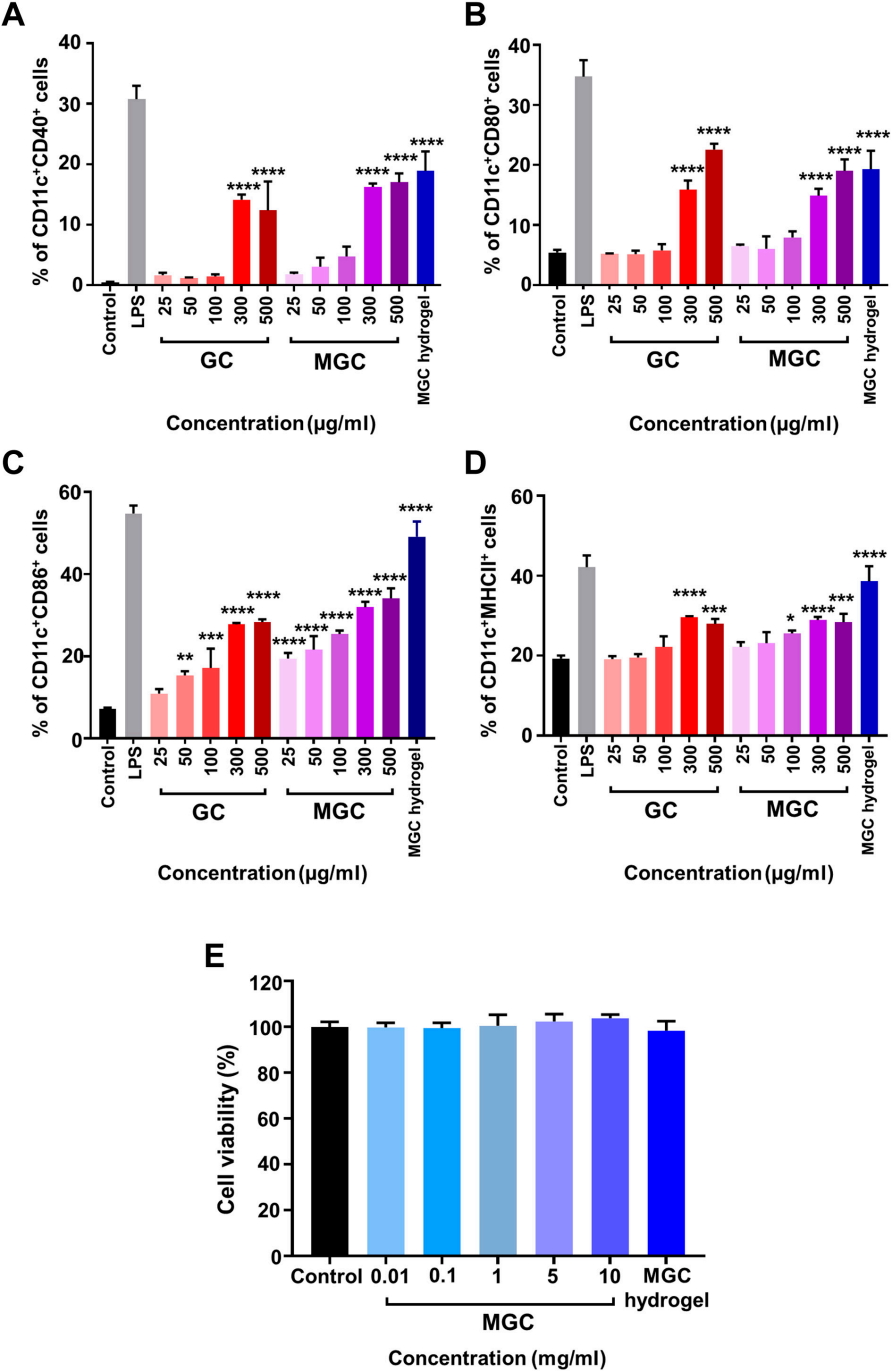
Adjuvant potency of GC, MGC, and MGC hydrogel and biocompatibility of MGC and MGC hydrogel. (A to D) The percentage of CD40^+^ (A), CD80^+^ (B), CD86^+^ (C), and MHCII^+^ (D) DCs. ANOVA was used for statistical analysis. Data are presented as means ± SD (*n* = 3; *P < 0.05, **P < 0.01, ***P < 0.001, and ****P < 0.0001). (E) Cytotoxicity of MGC and MGC hydrogel on L929 cells (*n* = 3).

Activation of DCs is an essential aspect of the adjuvant’s efficacy [43]. To evaluate the immunostimulatory effects of GC, MGC, and MGC hydrogel on DCs, we investigated their ability to modulate the surface expression of the costimulatory molecules CD40, CD80, CD86, and MHCII on DCs by flow cytometry analysis [44,45]. For the evaluation of the impact on immune cells in vitro, non-cross-linked MGC was utilized. In addition, to confirm the maintenance of immune-stimulatory capabilities even after forming the MGC hydrogel with non-cross-linked MGC, dried MGC hydrogel was added to RPMI 1640 medium. This facilitated the production of a decomposed hydrogel extraction solution containing non-cross-linked MGC. Subsequently, this extracted solution was utilized for evaluating immune activation. These costimulatory molecules coordinate key signals to drive T cell responses through the BMDCs. As expected, a strong enhancement of all surface expressions was observed. High-concentration treatment groups increased the percentages of DCs expressing CD40, CD80, CD86, and MHCII (Fig. 3A to D). The percentages of CD40, CD80, CD86, or MHCII^+^ DCs were dose-dependently increased by GC and MGC treatment. This increased levels of DCs expressing costimulatory molecules reached significance at the highest concentrations of GC and MGC tested than in the control. Specifically, in the MGC treatment group, up to 30-fold increases in CD40^+^ DCs and up to 4-fold increases in CD80^+^ and CD86^+^ DCs were observed compared to the control (Fig. 3A to C). Increases in MHCII^+^ DCs were at a lesser degree (Fig. 3D). The increase in DCs expressing costimulatory molecules was concentration dependent, with maximum responses observed at the highest concentrations. In addition, the percentage of DCs expressing these markers significantly increased in the MGC hydrogel extract treatment groups. The percentages of CD86^+^ and MHCII^+^ DCs significantly increased by 7-fold and 2-fold, respectively, compared to the control group (Fig. 3C and D).

### Biocompatibility of MGC and MGC hydrogels

To assess the effects of MGC and MGC hydrogels on cell viabilities, we conducted a CCK assay using L929 cell lines. As shown in Fig. 3E, both MGC and MGC hydrogel extracts did not exhibit significant toxicity. Cell viability remained above 99%, similar to that of the control group, at all MGC treatment concentrations. The MGC hydrogel extract group also exhibited a cell viability of around 98%, similar to previous reports [46,47].

### Degradation property of MGC hydrogel

**Fig. 4.**
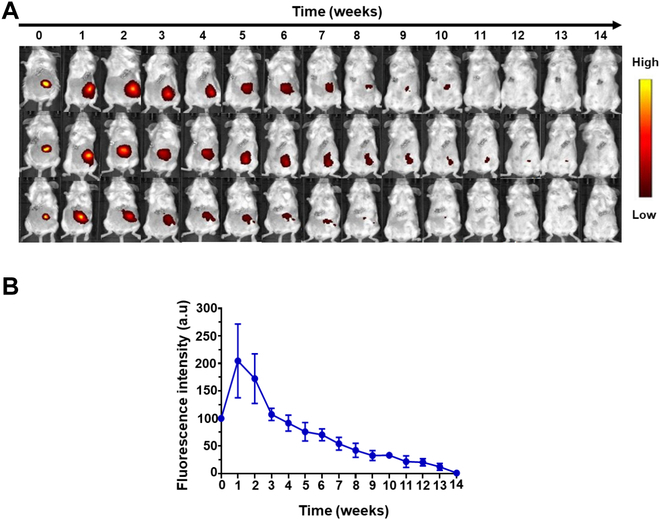
Degradation property of multifunctional MGC hydrogel. (A) Representative fluorescence IVIS imaging depicting the in vivo degradation profile. (B) Quantification of the in vivo degradation profile of Cy7-MGC hydrogel. Data are presented as means ± SD (*n* = 5). a.u., arbitrary units.

GC is commonly considered biocompatible and biodegradable, making it an ideal platform for biomedical applications [48–50]. It is well established that lysozyme, an enzyme present in vivo, cleaves the β-(1,4)-glycosidic bonds between *N*-acetylglucosamine (GlcNAc) and *N*-acetylmuramic acid units in the chitosan polymer chains [41,51,52]. To confirm the in vivo degradability of the hydrogel, we covalently conjugated a fluorescent dye (i.e., Cy7) with it. Fluorescent labeling provided a reliable and quantitative relationship between fluorescence reduction and hydrogel degradation. The hydrogel was then placed into the 4T1 tumor resection site of a female BALB/c mouse. The fluorescently labeled MGC hydrogel was monitored by IVIS imaging (Fig. 4A), and the data were quantified (Fig. 4B). A distinct decay of the fluorescence signal in vivo was observed from the hydrogels over the degradation time. However, when compared to Day zero, during the first and second weeks, the hydrogel’s fluorescence signal was approximately 1.5 to 2 times higher. Significant hydrogel degradation began around the sixth week, with complete absorption occurring by the 14th week.

### Release profile of MGC hydrogel

**Fig. 5.**
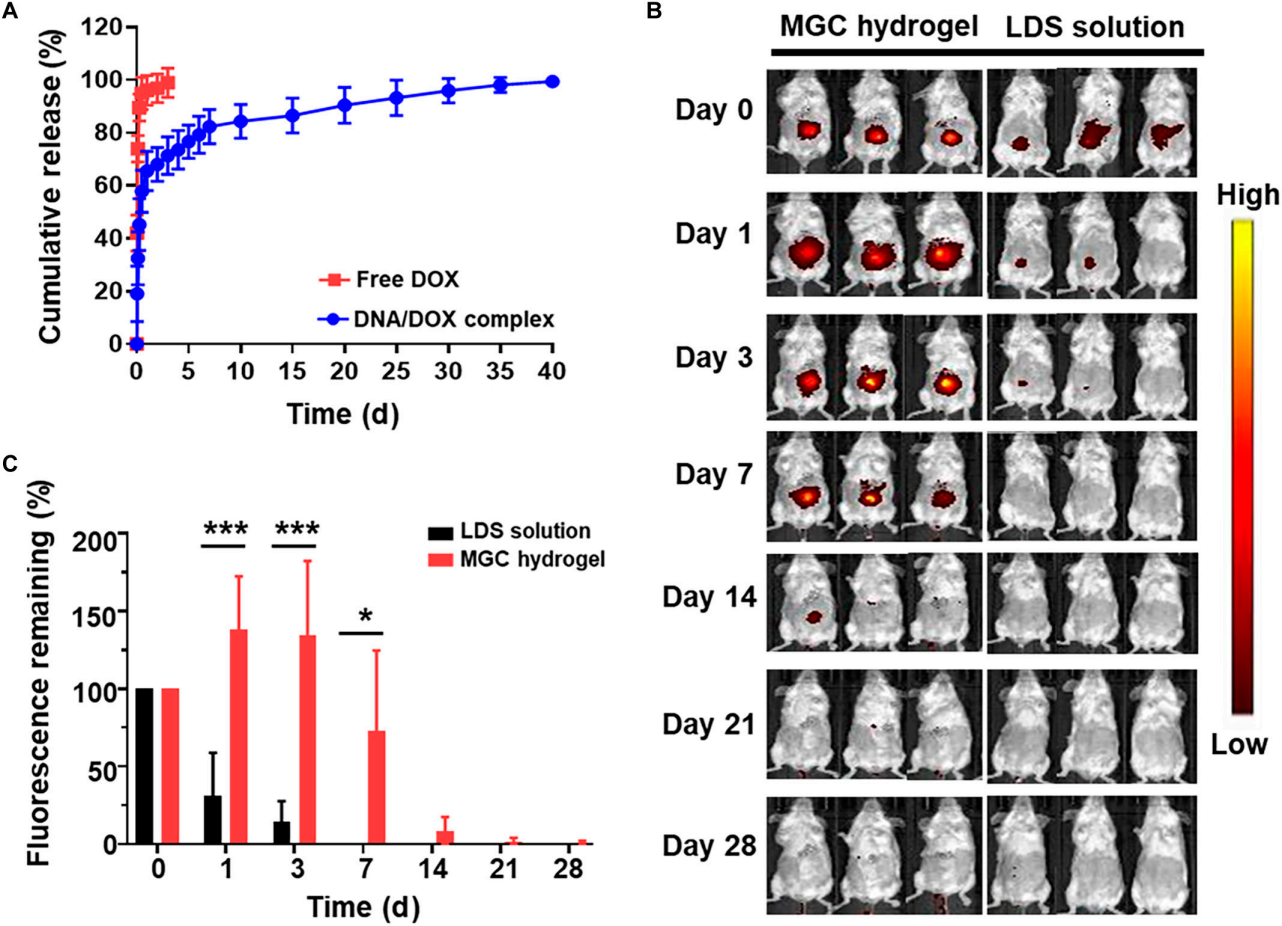
Release profile of multifunctional MGC hydrogel. (A) In vitro drug release profiles of MGC hydrogel loaded with DOX and the DNA/DOX complex. (B) Fluorescence IVIS imaging illustrating the in vivo release profile. (C) Quantitative analysis of fluorescence IVIS imaging of in vivo release profile. Data are presented as means ± SD (*n* = 5; *P < 0.05 and ***P < 0.001).

We quantified the encapsulated DNA/DOX complexes in MGC before investigating in vitro release (Fig. S5). In this study, we used DOX hydrochloride, known for its hydrophilic properties and widespread use in clinical practice. The hydrogel demonstrated high encapsulation efficiency, absorbing over 95% of the DNA/DOX complex. In addition, fluorescence imaging confirmed the robust fluorescence of the DNA/DOX complex within the hydrogel (Fig. S6). We then evaluated the in vitro release of DNA/DOX complexes and free DOX from MGC hydrogels (Fig. 5A). An immediate and complete release profile was evident within the first 12 h for the hydrogel containing free DOX. It was confirmed that the entirety of the DOX was released from the hydrogel within a span of just 3 d. Conversely, the hydrogels containing DNA/DOX complexes demonstrated an initial burst release within the first 3 d, followed by a sustained drug release that persisted for up to 40 d.

To complement this in vitro study, we examined the release kinetics of biologics in vivo. DNA labeled with the fluorescent nucleic acid dye, LDS 751, was administered to nontumor-bearing mice in solution or loaded in a scaffold and placed at the fourth mammary fat pad. The mice were assessed by fluorescence IVIS imaging (Fig. 5B), and the data were quantified (Fig. 5C). While approximately 80% of the signal decayed within 3 d of administration when using the fluorophore in solution, it took 28 d to achieve the same signal decay for the fluorophore loaded in the hydrogel. Over this time course, the signal for the fluorophore released from the MGC hydrogel was, on average, approximately 4-fold higher than the fluorophore in solution.

### In vitro ICD assay

**Fig. 6.**
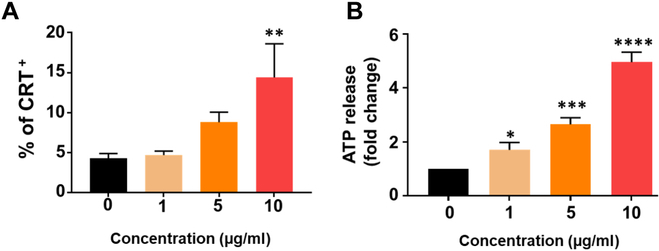
In vitro evaluation of ICD induced by DNA/DOX complex in 4T1-Luc2 cells. (A) The exposure of CRT on the cell surface was determined via flow cytometry. (B) released ATP levels in the culture medium after DNA/DOX complex treatment under various concentrations. Data are presented as means ± SD (*n* = 3; *P < 0.05, **P < 0.01, ***P < 0.001, and ****P < 0.0001).

Apart from its conventional use as a chemotherapeutic agent, DOX is recognized for inducing an immunological response through a unique cancer-killing mechanism called ICD [53]. We confirmed the induction of ICD by the DNA/DOX complex through the examination of CRT expression and release of ATP, which are DAMPs (Fig. 6). In response to treatment with different concentrations of DNA/DOX complex, 4T1-Luc2 cells underwent ICD, as indicated by the preapoptotic translocation of CRT. As shown in Fig. 6A, the percentage of CRT-positive cells increased dose-dependently. In addition, our results demonstrated that ATP release also increased within a dose-dependent manner in different cell groups (Fig. 6B). The expression level of DAMPs considerably increased, indicating that the DNA/DOX complex effectively induced ICD.

### Antitumor effect of drug-loaded MGC hydrogels

**Fig. 7.**
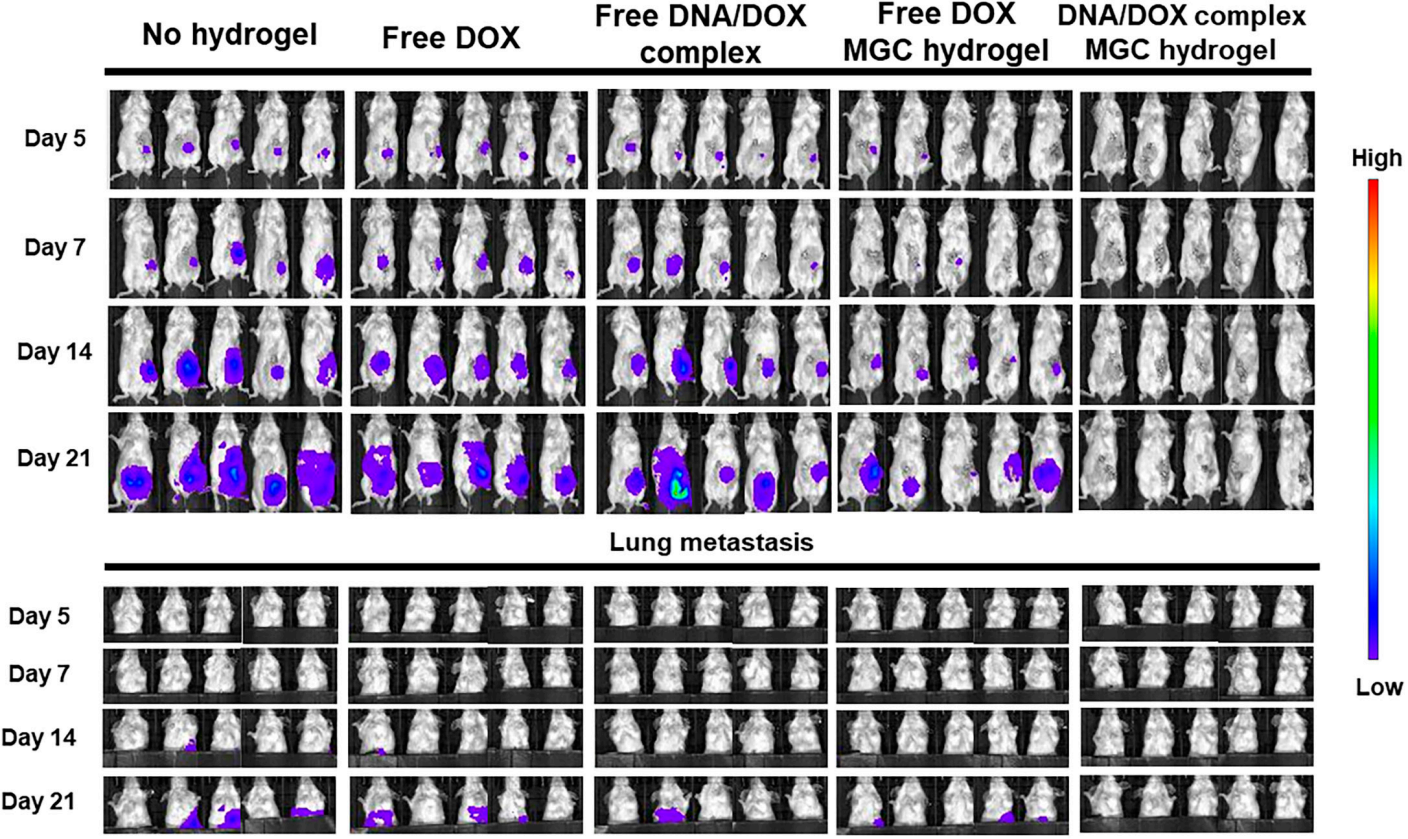
In vivo tumor suppression via multifunctional MGC hydrogel treatment in 4T1-Luc2 tumor model. IVIS imaging of 4T1-Luc2 cells was shown for all groups, describing, and illustrating tumor burden (*n* = 5).

To evaluate the suppression of tumor growth by the DNA/DOX complex-loaded MGC hydrogel, we conducted an in vivo tumor growth experiment. Female BALB/c mice were orthotopically inoculated with 4T1-Luc2 breast cancer cells in their fourth mammary fat pad. When tumors reached a volume of approximately 100 mm^3^, surgery was performed, intentionally leaving about 10% of the tumor tissue for simulating the incomplete removal of tumors during surgery. After tumor resection surgery, irregular cavities were formed at the surgical site. The MGC hydrogel, loaded with either the DNA/DOX complex or free DOX, was implanted into these cavities to fill the void space. The flexible and viscous nature of the MGC hydrogel allowed it to adapt to the irregular shape of the surgical site and effectively fill the cavities. Mice with comparable tumor sizes underwent surgical intervention, and those with similar residual tumor sizes were carefully selected for further experiments (Fig. S7). The mice were imaged using bioluminescent IVIS imaging, confirming the tumor sizes. As shown in Fig. 7, we demonstrated that local tumor recurrence was most effectively prevented when the DNA/DOX complex was administered through the hydrogel.

**Fig. 8.**
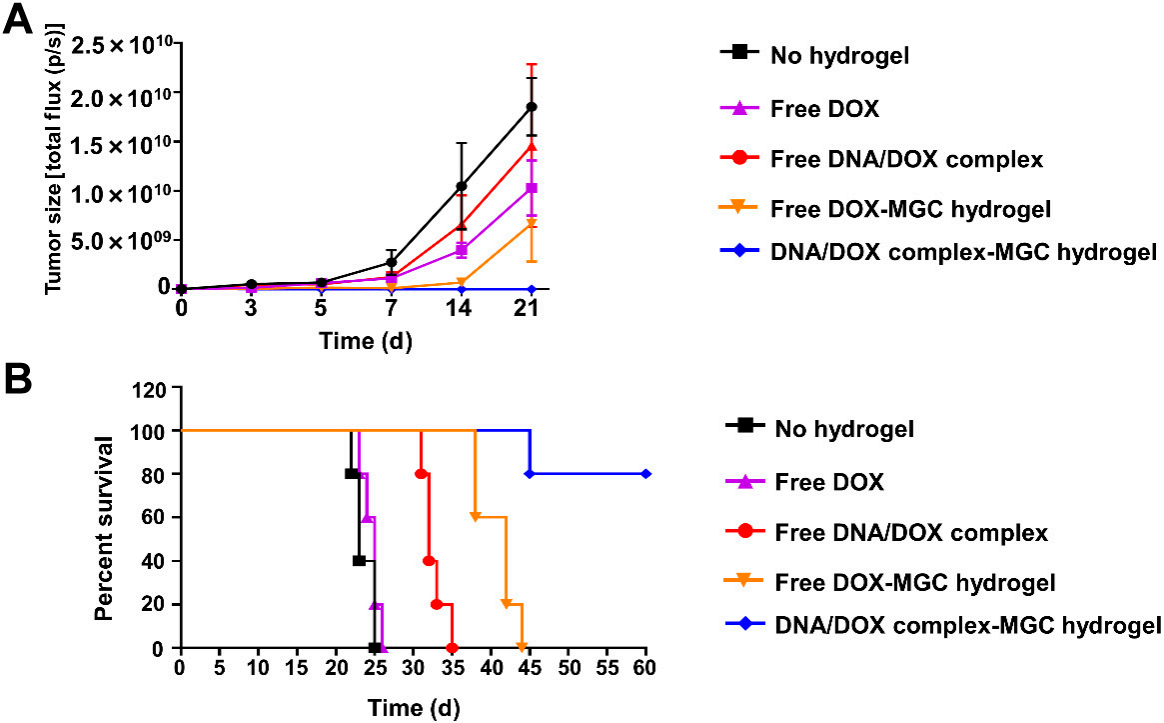
In vivo tumor suppression via multifunctional MGC hydrogel treatment in 4T1-Luc2 tumor model. (A) Mean change in tumor growth after treatment. (B) Survival after treatment in the 4T1-Luc2 tumor model. Data were presented as means ± SEM (*n* = 5).

The cancer cell signals in the IVIS images presented in Fig. 7 were quantitatively analyzed and depicted in Fig. 8A and Fig. S8. The control group, which did not receive hydrogel treatment, exhibited increased tumor signals over time, high rates of tumor recurrence, and a 100% mortality rate within 25 d. Moreover, the group treated with free DOX and free DNA/DOX complex alone showed no significant therapeutic effect, as their tumor growth rates were similar to those of the control group. While the free DOX-loaded hydrogel-treated group initially demonstrated a decrease in tumor growth rate, this rate increased after approximately 14 d. However, the group treated with the MGC hydrogel loaded with DNA/DOX complex consistently effectively inhibited tumor growth. In addition, this group had the lowest metastasis rate to the lungs when compared to the other groups (Fig. 7). Subsequently, we evaluated the survival rate of the mouse breast tumor model following treatment, as illustrated in Fig. 8B. Although the MGC-hydrogel-treated group loaded with free DOX demonstrated improved survival compared to the control and free DOX-treated groups, the MGC-hydrogel-treated group loaded with DNA/DOX complex exhibited the longest survival rate among all groups. Notably, this group maintained 80% survival rate for approximately 60 d.

In summary, compared to treatments with MGC hydrogels containing free DOX or free DOX alone or free DNA/DOX complex alone, treatment with hydrogels containing DNA/DOX complexes showed greater efficacy in inhibiting tumor recurrence and metastasis, significantly prolonging the survival of mice. As evidenced in the orthotopic breast tumor model, we posit that the immune-cell-activating properties and sustained local release mechanism of MGC hydrogels played a crucial role in triggering the antitumor immune response, effectively suppressing tumor growth and metastasis. We plan to conduct more specific in vivo immunological assays in future studies to validate these findings further and elucidate the underlying mechanisms.

### Immune response by drug-loaded MGC hydrogels in vivo

**Fig. 9.**
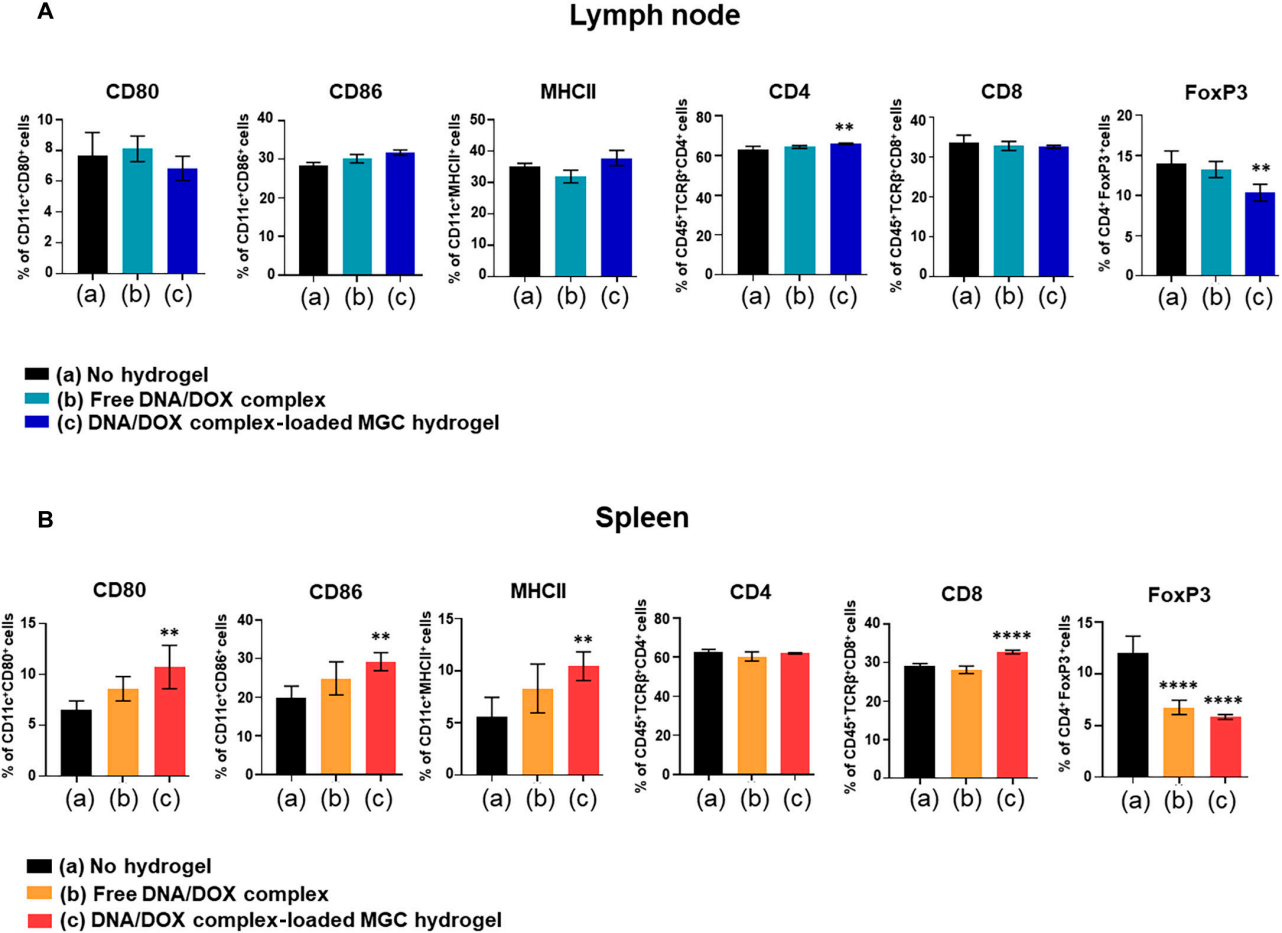
In vivo activation of immune cells response to combined treatment of the MGC hydrogel with DNA/DOX complex. (A) Percentage of activated DCs (CD80^+^, CD86^+^, and MHCII^+^) and T cells (CD4^+^, CD8^+^, and FoxP3^+^) in the lymph node after treatments. (B) Percentage of activated DCs (CD80^+^, CD86^+^, and MHCII^+^) and T cells (CD4^+^, CD8^+^, and FoxP3^+^) in the spleen after treatments. Data are presented as means ± SD (*n* = 5; **P < 0.01 and ****P < 0.0001).

To investigate the effect of the DNA/DOX complex-loaded MGC hydrogel treatment on immune cell activation, we analyzed the activation markers of DCs and T cells extracted from the lymph node and spleen, 10 d after treatment, in a 4T1 mouse tumor model (Fig. 9). The results showed that the DNA/DOX complex-loaded MGC hydrogel treatment significantly increased the number of DCs. We further assessed the maturation of DCs by analyzing the expression levels of CD80, CD86, and MHCII. The expression of CD80, CD86, and MHCII on DCs in lymph node did not show any significant difference between the DNA/DOX complex-loaded MGC hydrogel group and the control group (Fig. 9A). However, significant increases in all markers were observed in the MGC hydrogel group for DCs in the spleen (Fig. 9B). These results suggest that the DNA/DOX complex-loaded MGC hydrogel can effectively induce DC maturation. The DCs elicited a robust adaptive antitumor response, as evident from the T cell compartment observed 10 d after MGC hydrogel treatment. The expression of CD8 in the lymph nodes did not exhibit any significant differences between the DNA/DOX complex-loaded MGC hydrogel group and the control group (Fig. 9A). However, in the MGC hydrogel group, the expression of CD4 and FoxP3 on T cells showed a significant decrease compared to the control group. In addition, the results indicated a reduction in the number of CD4^+^FoxP3^+^ T cells and an increase in the number of CD8^+^ T cells expressing activation markers in the spleen (Fig. 9B). These findings demonstrate the effective induction of DC and T cell activation in vivo by the MGC hydrogel treatment.

## Discussion

This study introduces an innovative and versatile MGC hydrogel system designed as a local drug delivery platform aimed at enhancing postsurgical cancer treatment by modulation of antitumor immunity. We successfully confirmed the methacrylation of GC, leading to the formation of a robust MGC hydrogel with a well-defined structure (Fig. 2). The presence of macroporous characteristics in the hydrogel validates the successful methacrylation of GC and underscores its suitability for controlled release applications. GC, a natural polysaccharide composed mainly of glucosamine with a high cationic charge density, functions as an immunoadjuvant [54]. It possesses GlcNAc carbohydrate units that can interact with the macrophage mannose receptor, specifically recognizing mannose- and GlcNAc-containing glycoproteins [55]. These carbohydrate units enable GC to be recognized by pattern recognition receptors (PRRs) present on the cell membrane of DCs [56]. DCs express various PRRs, including Toll-like receptors and other PRRs [57]. The recognition of these patterns by PRRs initiates intracellular signaling pathways within DCs, resulting in their activation. In addition, the recognition of specific molecular patterns by PRRs on DCs triggers intracellular signaling cascades, including the activation of nuclear factor kappa-light-chain-enhancer of activated B cells (NF-κB) and mitogen-activated protein kinase pathways, known to be involved in DC maturation and regulation of immune responses [31]. Therefore, the presence of GC in the MGC hydrogel extracts activates DCs through the recognition of its carbohydrate units by PRRs, providing a rationale for the observed immune activation in vitro. These results indicated that the MGC hydrogel effectively induced DC maturation and provided an opportunity for initiating T cell responses.

A comprehensive assessment of the MGC hydrogel system’s efficacy was conducted through both in vivo and in vitro testing, serving as a crucial component of this study. These evaluations aimed to demonstrate the system’s ability to prevent tumor recurrence and eliminate distal metastases, thus emphasizing the potential of this drug delivery platform. MGC and MGC hydrogels’ effects on cell viabilities revealed encouraging results. Both MGC and MGC hydrogel extracts demonstrated nonsignificant toxicity, with cell viabilities consistently above 99%, comparable to the control group (Fig. 3). These results align with previous reports [46,47], suggesting that MGC hydrogel is biocompatible and noncytotoxic, highlighting its suitability for immunological applications. The biocompatibility of the hydrogel is a critical consideration when assessing its potential for in vivo and human body applications. The biodegradability of MGC hydrogel is a pivotal characteristic for its in vivo applications. Our study confirmed the in vivo degradability of the hydrogel by covalently conjugating a fluorescent dye to it. The observation of a decline in fluorescence signal over time in vivo indicated that substantial hydrogel degradation commenced at 6 weeks, with complete resorption achieved at 14 weeks (Fig. 4). However, when compared to day 0, during the first and second weeks, the hydrogel’s fluorescence signal was approximately 1.5 to 2 times higher. This phenomenon is believed to be attributed to the properties of cyanine fluorescent dyes, which significantly increase fluorescence when they bind to DNA within the body [58]. Furthermore, when Cy7-labeled MGC hydrogels are implanted, body fluids cause the hydrogels to swell and expand. This swelling potentially enhances the hydrogels’ interaction with biomolecules, leading to an amplified fluorescence signal. However, comprehensive studies are required to thoroughly understand this process. This extended degradation profile is advantageous for sustaining drug delivery, allowing for prolonged therapeutic effects at the intended site. It addresses a critical aspect of controlled drug release, ensuring that therapeutic agents are released gradually, aligning with treatment needs. The release profile of the MGC hydrogel, engineered for the localized and extended release of bioactive components, is a fundamental aspect of this study.

Encapsulation of DOX within hydrogels presents a significant challenge due to its relatively low molecular weight and hydrophilic properties leading not to an initial burst release but to an immediate and complete release, which poses a considerable challenge [59]. To overcome these obstacles, we used a strategy involving the intercalation of DOX into DNA, yielding DNA/DOX complexes [60,61]. The formation of these anionic DNA/DOX complexes facilitates the encapsulation of DOX within the hydrogel matrix using electrostatic interactions with the cationic GC hydrogel. This approach results in sustained DOX release and enhanced stability of the encapsulated drug, as reported in previous studies [60,62]. The release profile of MGC hydrogels was intentionally designed to incorporate an initial burst release, followed by an extended release at a lower rate (Fig. 5A). This strategy serves the dual purpose of delivering a high drug concentration rapidly to the tumor site immediately after resection, while also ensuring a continuous presence of the therapeutic agent for prolonged efficacy. In vitro release studies demonstrated that the hydrogel extends the release of DNA/DOX complexes compared to free DOX. Free DOX exhibited an immediate and complete release within 12 h, whereas the hydrogel containing DNA/DOX complexes displayed an initial burst release, followed by sustained release for up to 40 d. The prolonged and sustained release of the DNA/DOX complex is hypothesized to result primarily from the hydrolysis of DNA, although further investigation is required to fully understand the specific drug release mechanisms [63–65]. This indicates that DNA/DOX complexes within the hydrogel are more conducive to achieving a sustained release profile.

In vivo studies supported these findings, as the release of DNA labeled with a fluorescent dye showed an extended duration within the MGC hydrogel in comparison to solution-based delivery (Fig. 5B). As is widely acknowledged, the fluorescence of LDS 751 increased by up to 20-fold when bound to DNA, offering a sensitive and effective means to monitor DNA-bound dyes across diverse experimental contexts [66]. Consistent with the findings presented in Fig. 4, a higher fluorescence signal was observed in the MGC hydrogel on the first day after implantation compared to immediately after injection. This increased signal is attributed to the hydration of the MGC hydrogel by body fluids, resulting in its swelling. Such swelling enhances the accessibility of the LDS 751 binding sites within the DNA structure, thereby facilitating a stronger interaction between the dye and DNA. In addition, the in vivo microenvironment of the MGC hydrogel, influenced by factors like ionic strength and pH, may also affect the binding dynamics of LDS 751 to DNA. Further studies are required to fully elucidate the mechanisms underlying these observations. The MGC hydrogel exhibited an approximately 9-fold longer extension than solution-based delivery over 28 d. The release rates in vitro exhibited prolonged kinetics relative to those in vivo, as expected for an environment that is more physiologically relevant than sink conditions. These data confirm that the MGC hydrogel can substantively extend the local release of immunomodulatory compounds relative to local delivery of the same compounds in solution. These extended and slowly released behaviors of the therapeutic agents offer the opportunity to prevent systemic and acute toxicity effects.

One of the remarkable features of DOX, aside from its traditional chemotherapeutic role, is its ability to trigger an immunological response through ICD [67]. This unique mechanism of cancer cell elimination was confirmed through the examination of key indicators, including the expression of CRT and the release of DAMPs such as ATP. Our results clearly demonstrated that the DNA/DOX complex effectively induced ICD in breast cancer cell, as evidenced by the dose-dependent increase in CRT-positive cells and increased ATP release (Fig. 6). These findings underscore the potential of the DNA/DOX complex as an effective inducer of ICD. Furthermore, the successful local release of a DNA/DOX complex, facilitated by the advantages of the MGC hydrogel, provides targeted and efficient treatment against tumor growth and dissemination.

The MGC hydrogel loaded with the DNA/DOX complex consistently and effectively suppressed tumor growth (Figs. 7 and 8). In addition, this treatment group exhibited the lowest lung metastasis rate among all groups. The survival analysis further highlighted the superiority of the DNA/DOX complex-loaded MGC hydrogel, with the longest survival rate, maintaining an impressive 80% survival rate for 60 d. These results emphasize the potential of the MGC hydrogel as an efficient carrier for the DNA/DOX complex, ensuring sustained therapeutic effects and enhanced tumor growth suppression. One of the key strengths of the MGC hydrogel system lies in its ability to create a conducive microenvironment for immune cells within the tumor site (Fig. 9). This microenvironment promotes the maturation and activation of immune cells, thereby enhancing antitumor immune responses. Such an approach aligns with the growing interest in harnessing the immune system’s natural defenses to combat cancer [68]. The MGC hydrogel loaded with the DNA/DOX complex significantly increased the number of DCs, with the spleen showing substantial DC maturation marked by increased expression of CD80^+^, CD86^+^, and MHCII^+^. This suggests that the DNA/DOX complex-loaded MGC hydrogel effectively induces DC maturation, a critical step in activating the adaptive antitumor response. The T cell compartment analysis revealed intriguing findings, with a significant decrease in the number of CD4^+^FoxP3^+^ regulatory T cells and an increase in the number of CD8^+^ T cells expressing activation markers in the spleen. These results indicate the potential of the MGC hydrogel treatment to induce DC and T cell activation in vivo.

In summary, the biomaterial-based drug delivery system developed in this study, when integrated with cancer immunotherapy, represents a significant advancement in oncology treatment. The findings underscore the potential to improve patient prognosis by harnessing the unique features and innovations offered by the MGC hydrogel system. This approach not only holds promise for enhancing the effectiveness of cancer treatment but also highlights the importance of multidisciplinary research and innovation in addressing complex medical challenges. Future investigations and clinical studies may further elucidate the full clinical potential of this innovative drug delivery platform.

### Conclusion

In this study, we developed a novel multifunctional MGC hydrogel system as a local drug delivery platform for modulating antitumor immunity to enhance tumor immunotherapy. We further demonstrated the efficacy of this system through in vivo and in vitro testing. GC at a defined concentration induced maturation of BMDCs, supporting its potential role as an adjuvant for enhancing immunity in cancer immunotherapy. Through harnessing the advantages of the immune-activating properties of GC, we fabricated MGC hydrogel and successfully demonstrated that local release of the DNA/DOX complex in the MGC hydrogel effectively prevented tumor recurrence and eliminated distal metastases. Notably, the MGC hydrogel developed in this study effectively induced the inhibition of tumor recurrence and metastasis after surgery. This biomaterial-based drug delivery system, combined with cancer immunotherapy, can potentially enhance oncology treatment outcomes and improve patient prognosis.

## Ethics Declarations

Ethics approval and consent to participate: All procedures for animal experiments in this study were approved by the IACUC of Sungkyunkwan University School of Medicine (approval number: SKKUIACUC2023-02-16-1) and complied with the guidelines for the care and use of laboratory animals. This university is accredited by the guidelines of the National Research Council’s Guide for the Care and Use of Laboratory Animals. Consent for publication: Not applicable.

## Data Availability

The datasets used and analyzed during the current study are available from the corresponding author on reasonable request.
