# Supplementary figures and images for: Enhanced Postsurgical Cancer Treatment Using Methacrylated Glycol Chitosan Hydrogel for Sustained DNA/Doxorubicin Delivery and Immunotherapy
Source: Biomater Res. 2024 Mar 23;28:0008. doi: 10.34133/bmr.0008 (PMC10964224; doi:10.34133/bmr.0008)

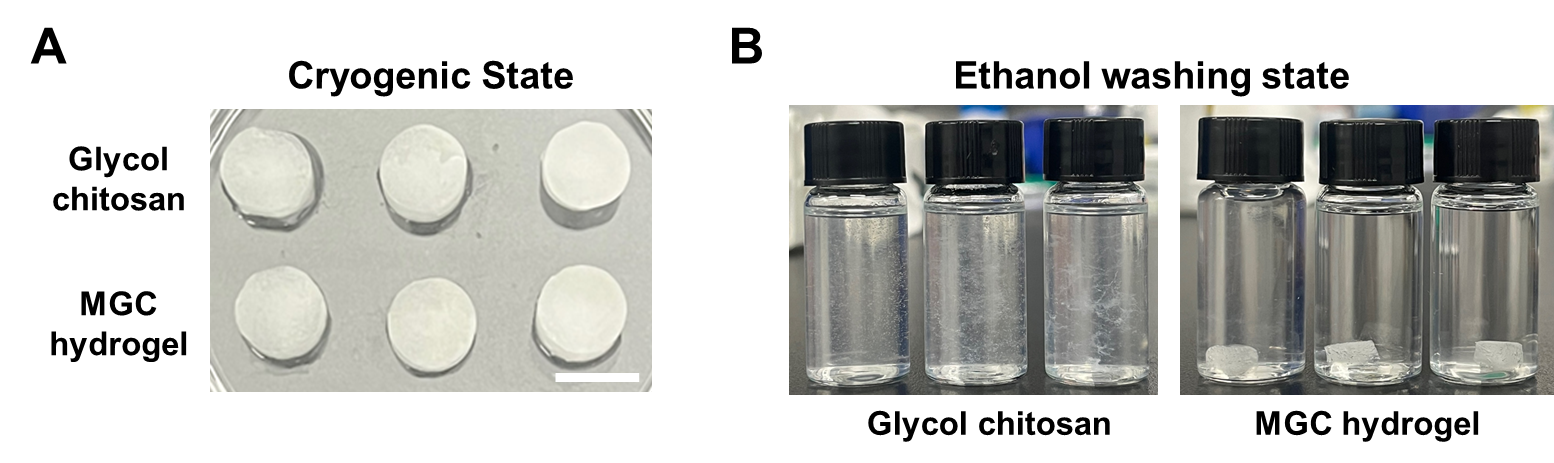

Supplement: Supplementary 1 — Figs. S1 to S8 [file bmr.0008.f1.zip › Fig.S1.tif]

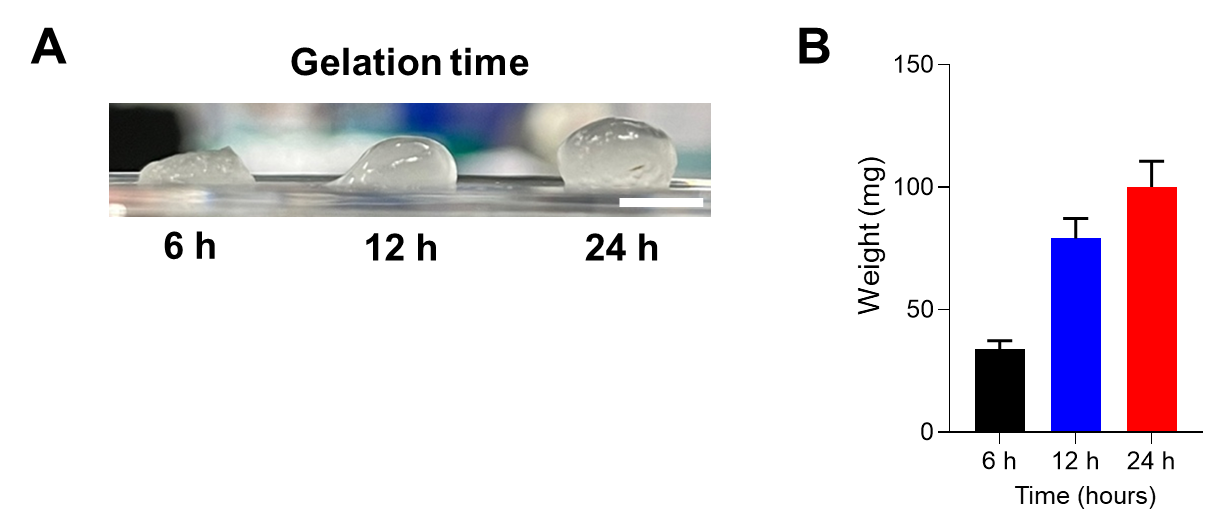

Supplement: Supplementary 1 — Figs. S1 to S8 [file bmr.0008.f1.zip › Fig.S2.tif]

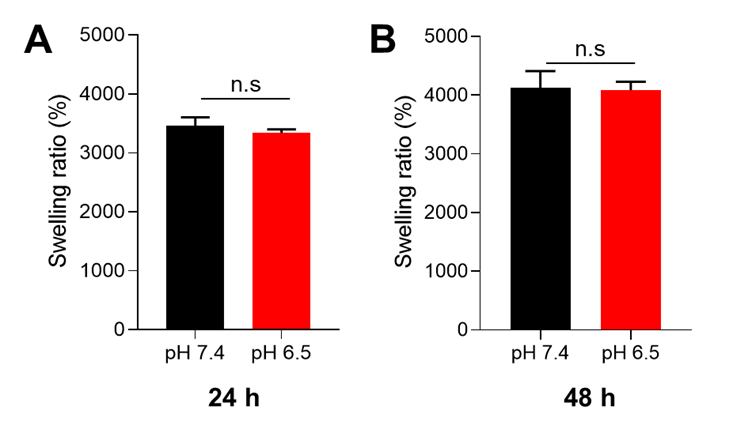

Supplement: Supplementary 1 — Figs. S1 to S8 [file bmr.0008.f1.zip › Fig.S3.tif]

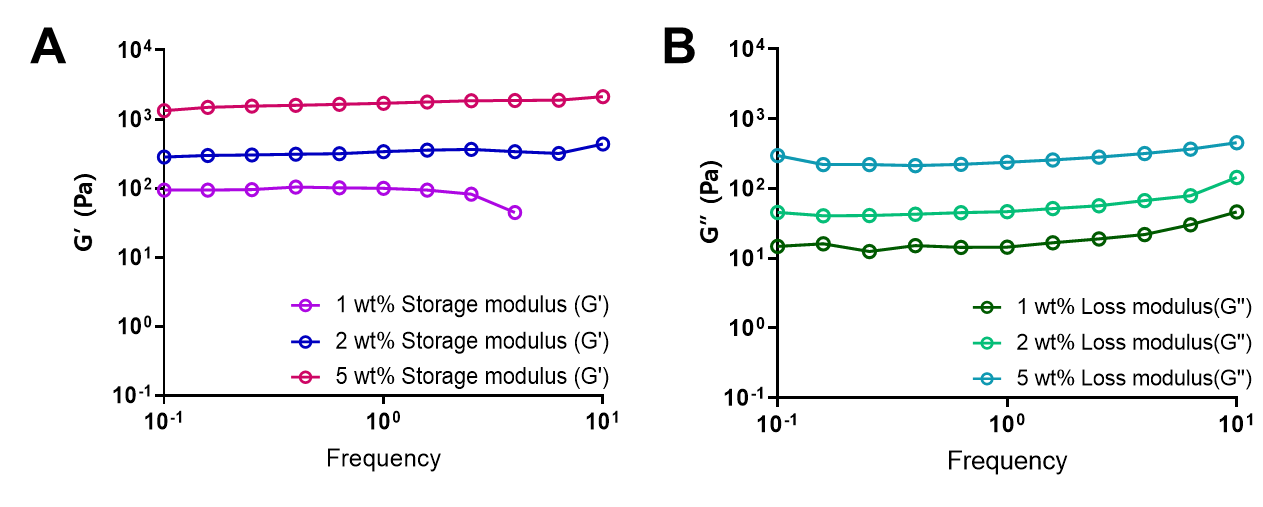

Supplement: Supplementary 1 — Figs. S1 to S8 [file bmr.0008.f1.zip › Fig.S4.tif]

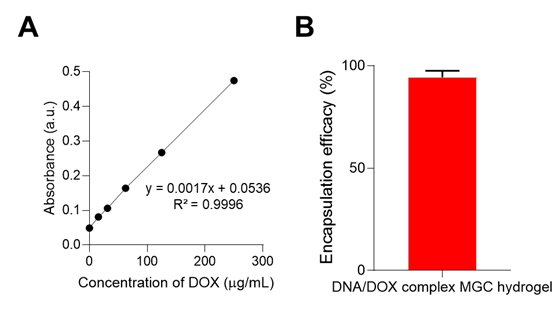

Supplement: Supplementary 1 — Figs. S1 to S8 [file bmr.0008.f1.zip › Fig.S5.tif]

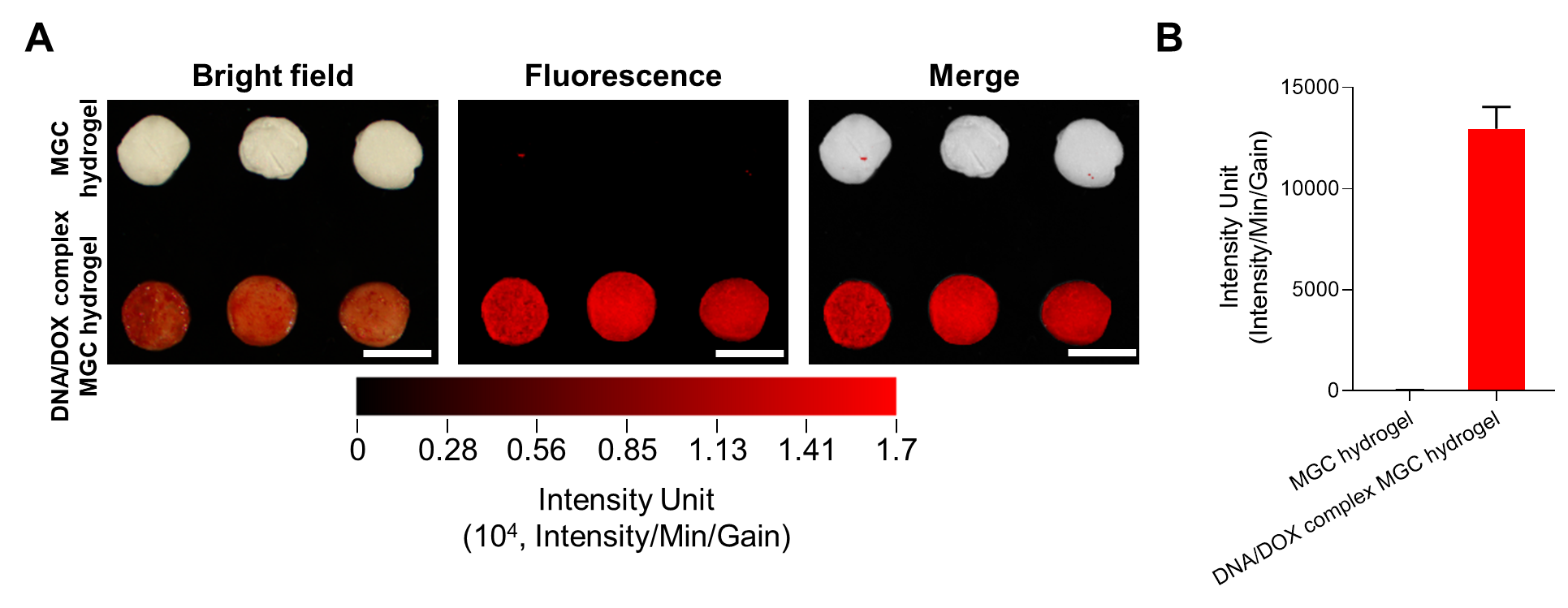

Supplement: Supplementary 1 — Figs. S1 to S8 [file bmr.0008.f1.zip › Fig.S6.tif]

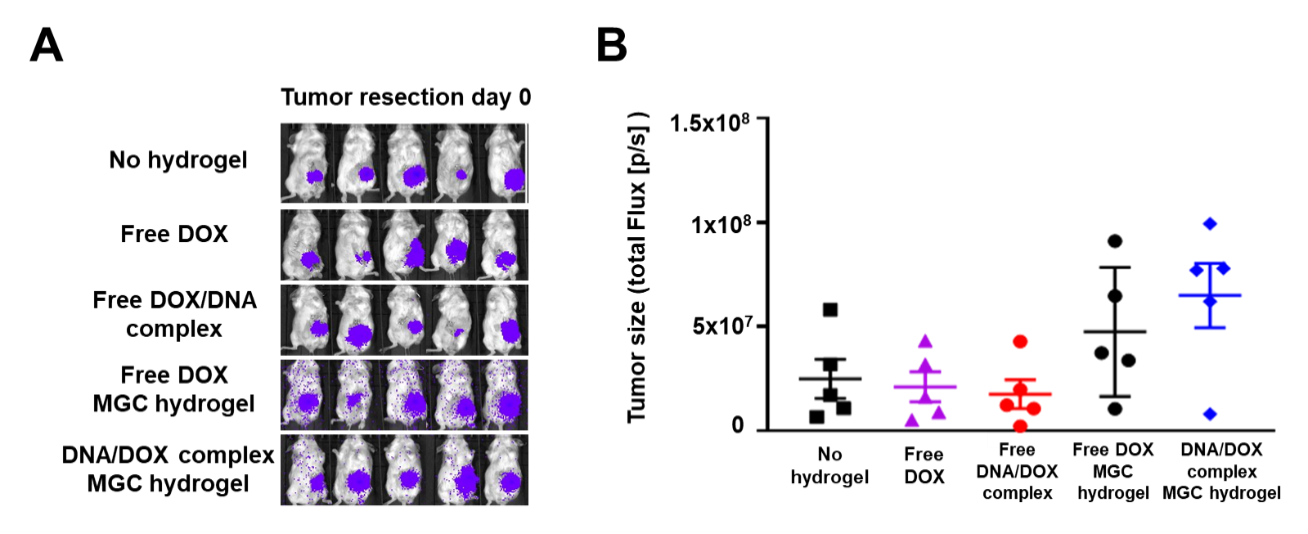

Supplement: Supplementary 1 — Figs. S1 to S8 [file bmr.0008.f1.zip › Fig.S7.tif]

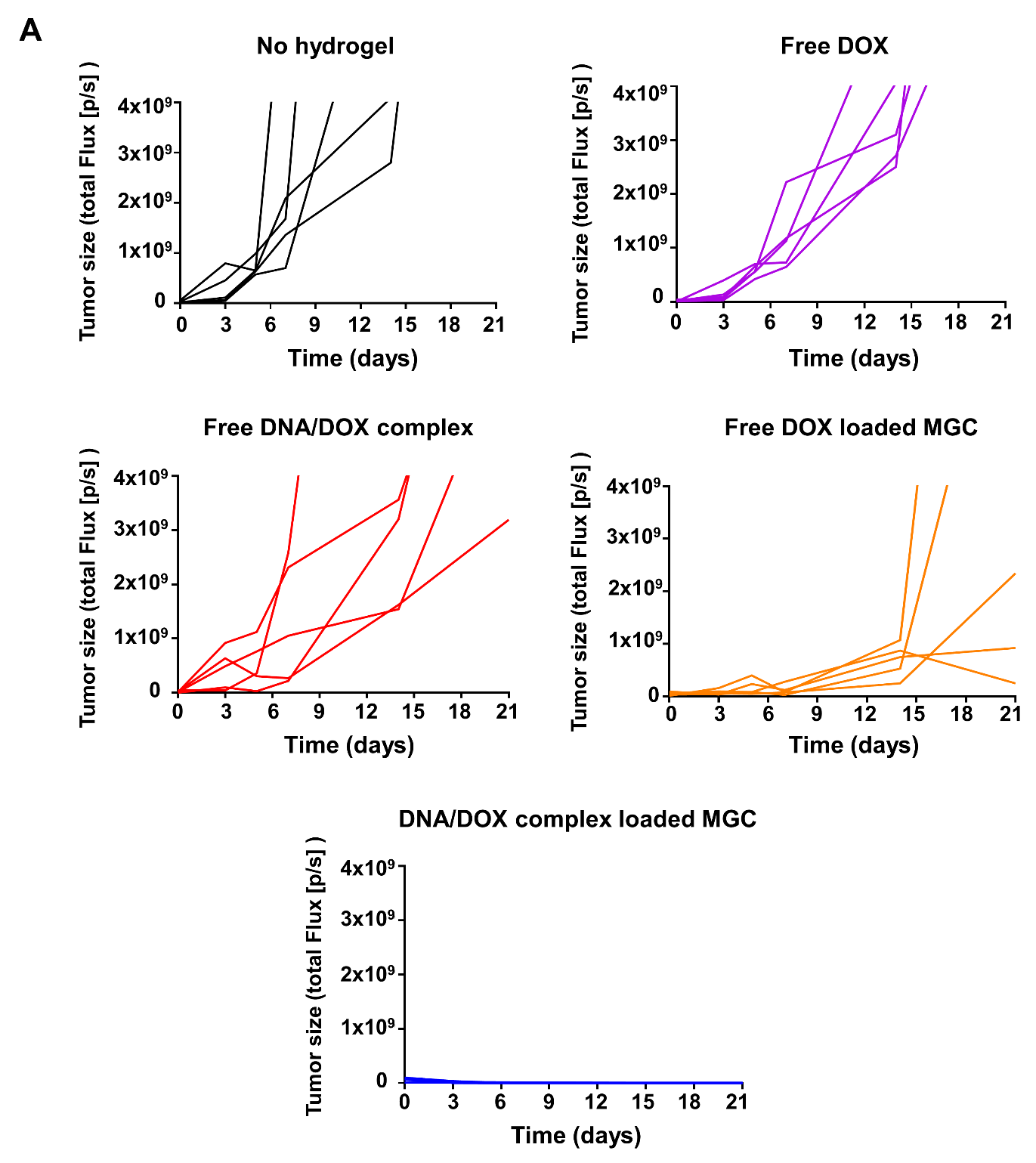

Supplement: Supplementary 1 — Figs. S1 to S8 [file bmr.0008.f1.zip › Fig.S8.tif]

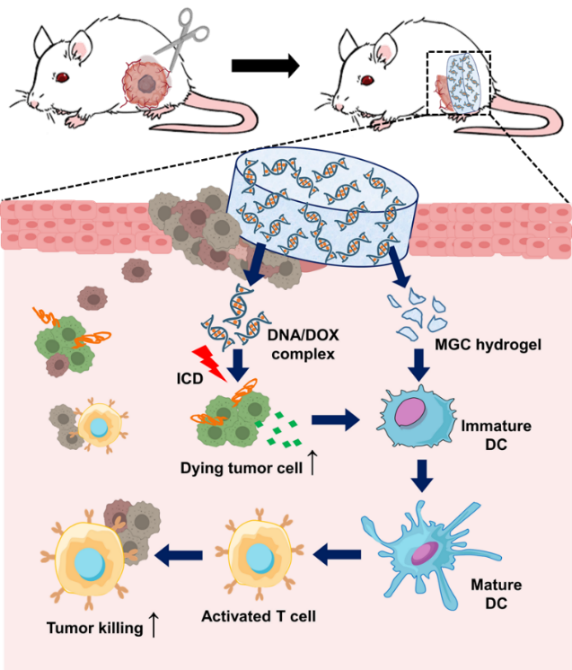

Supplement: Supplementary 1 — Figs. S1 to S8 [file bmr.0008.f1.zip › Graphic abstract.tif]
